# *TP53* Mutations in Chagasic Megaesophagus

**DOI:** 10.3390/cimb47040229

**Published:** 2025-03-27

**Authors:** Aparecida Perpetuo Fedossi Silveira, Ricardo Quiterio Sartori, Lilian Castiglioni, Heidi Lacerda Alves da Cruz, Eumildo de Campos Junior, Cinara Cássia Brandão, Luiz Carlos de Mattos

**Affiliations:** 1 Laboratório de Imunogenética, Departamento de Biologia Molecular, Faculdade de Medicina de São José do Rio Preto (FAMERP), São José do Rio Preto 15090-000, SP, Brazil; 2 Instituto de Biociências, Letras e Ciências Exatas (IBILCE), Universidade Estadual Paulista Júlio de Mesquita Filho (UNESP), São José do Rio Preto 15054-000, SP, Brazil; 3 Laboratory of Immunopathology Keizo Asami, Universidade Federal de Pernambuco (UFPE), Recife 50670-901, PE, Brazil; 4 Departamento de Cirurgia do Hospital de Base, Fundação Faculdade Regional de Medicina de São José do Rio Preto (FUNFARME), São José do Rio Preto 15090-000, SP, Brazil

**Keywords:** Chagas disease, esophageal achalasia, megaesophagus, esophageal squamous cell carcinoma, *TP53* gene

## Abstract

Patients carrying Chagasic megaesophagus (CME) are at high risk for esophageal carcinoma. We aimed to investigate mutations in the *TP53* in patients carrying CME. Blood samples from 114 patients with Chagas disease (CD) were used. The samples were subjected to PCR-SSCP analysis and DNA sequencing in exons 5 and 7 of the TP53 gene. We observed mutations in the exon 5 codon 184 (GAT > AAT) in 14.8% of G1 (11/74), 10% of G2 (4/40), and 5% of G3 (2/40). We also observed the codon 185 mutation (AGC > AGG) in 14.8% of G1 (11/74), 10% of G2 (4/40), and 7.5% of G3 (3/40). Regarding Exon 7, a mutation (G > T) was observed in the intronic region in 2.7% of G1 (2/74), 7.5% of G2 (3/40), and none of G3 (0/40). Our study showed, for the first time, simultaneous mutations at codons 184 and 185 of the *TP53* gene in patients with CME and Chagasic patients without megaesophagus. More studies are needed to assess whether the simultaneous presence of mutations at codons 184 and 185 increases the risk of developing esophageal carcinoma in these patients.

## 1. Introduction

Chagas disease (CD) is endemic to Latin America but affects about 7 million people worldwide [1]. In its chronic phase of the disease, *Trypanosoma cruzi* lodges mainly in the heart tissue and the digestive tract. Among the most common clinical manifestations of this phase, cardiomyopathy stands out, affecting about 30% of patients. The digestive forms, such as megaesophagus and megacolon, occur in approximately 5 to 10% of individuals [2]. Chagasic megaesophagus (CME) is a consequence of a motor disorder known as achalasia, characterized by the destruction or loss of the intramural nervous plexus of the esophagus. This event causes impaired peristalsis and loss of opening of the lower esophageal sphincter (LES) in response to swallowing. These changes cause the retention of food inside the organ, causing its progressive dilation and the appearance of esophagitis, acanthosis, parakeratosis, and precancerous lesions [3].

In Brazil, the only proven etiological factor of achalasia and megaesophagus is CD, as *T. cruzi* is the agent responsible for destroying the myenteric plexus of the esophagus [4]. The parasite triggers a local inflammatory process by infecting esophageal myenteric cells, which impairs nerve impulse transmission and normal LES relaxation [5].

Patients with CME have a 33 times greater risk of developing esophageal carcinoma (EC) than the general population [6]. Tumor development is related to prolonged contact of food with the esophageal mucosa due to food stasis, high bacterial growth, chemical irritation, and chronic esophagitis (CE) [3]. Esophageal squamous cell carcinoma (ESCC) is the most common form of esophageal cancer, and it has a high mortality rate. In Brazil, esophageal cancer is the sixth most frequent among men, the fifteenth most frequent among women, and the eighth most frequent worldwide. According to statistics, it caused about 8716 deaths, estimated at 11,390 new cases [7]. The development of ESCC is a multifactorial process, as it is associated with several environmental, pathological, and genetic factors. The main risk factors include alcohol and tobacco consumption, caustic lesions in the esophagus, consumption of hot drinks, nutritional deficiencies, older age, male gender, black race, tylose, papillomavirus infection, achalasia, and CD [8]. The development of ESCC is a progressive process and has several steps. Some studies suggest that the sequence of morphological alterations involved in this process begins with hyperplasia of the epithelial cells of the basal layer, followed by low to high-grade dysplasia, carcinoma in situ, and invasive carcinoma, accompanied by numerous genetic alterations. Among them, aneuploidies, allelic deletions, oncogene activation, and tumor suppressor gene inactivation stand out [8,9].

About 60% of human cancers have mutations in the TP53 gene, a tumor suppressor located on chromosome 17 (17p13.1) [10]. This gene encodes a 393 amino acid nuclear phosphoprotein called p53, which acts as a transcription factor responsible for gene activation in cell cycle control and apoptosis [10,11]. The regulation of these genes coordinated by p53 results in antiproliferative effects, preserving the integrity of the genome, while the loss of its function favors a rapid accumulation of multiple genetic alterations leading to malignant phenotypes [9]. In EC, 92% of TP53 mutations are located between exons 5 and 8, occurring most commonly in the p53 DNA-binding domain, between amino acid residues 102–292, with hot spots at codons 175, 176, 248, 273, and 282. Most of these are point mutations, including transitions and transversions, missense mutations, in which a single base exchange leads to an amino acid substitution, which may result in a non-functional protein, frameshift mutations due to the insertion or deletion of bases altering the reading frame, and nonsense type, whose formation of stop codons leads to the production of an incomplete protein [10,12]. Additionally, p53 gain-of-function mutations are located in *TP53* hot spots and *TP53* DNA-binding, which favor cancer development [10]. This study aimed to investigate mutations in the *TP53* gene in patients with CME since in such premalignant lesions, these findings are scarce.

## 2. Materials and Methods

### 2.1. Study Design and Participants

A total of 114 patients with CD, confirmed by serological and molecular tests, were selected: 74 patients with megaesophagus alone or associated with megacolon (Group 1) and 40 patients with the indeterminate form of CD or with Chagas heart disease but without Megaesophagus (Group 2). Additionally, 40 healthy individuals who were blood donors with negative serology for CD (Group 3) were used.

Except for blood donors, who were obtained from the Blood Center of Hospital de Base/Fundação Faculdade Regional de Medicina de São José do Rio Preto (Funfarme), SP, Brazil, all patients were chosen at the Surgery Outpatient Clinic of Funfarme, SP, Brazil, in 2011. The Faculdade de Medicina de São José do Rio Preto (FAMERP) Research Ethics Committee (CEP: 3,463,755) accepted the trial, and each patient signed an informed permission form. According to the previous classification [11], the cases of megaesophagus presented varying degrees of esophageal dilation, being classified as Grade I (*n* = 4), Grade II (*n* = 28), Grade III (*n* = 9), and Grade IV (*n* = 8). In one individual, it was not possible to distinguish between Grade II/III, and in 19 individuals, it was not possible to distinguish between Grade III/IV.

The present study was prospective, and patients with any type of cancer were excluded from the study. The inclusion criteria were considered: positive laboratory diagnosis of CD in the chronic phase at the time of the study, presenting the indeterminate form of the disease or cardiac or digestive clinical manifestations according to the II Brazilian Consensus on CD.

### 2.2. Procedures

Genomic DNA was extracted from peripheral blood using the DNeasy Blood and Tissue Kit (Qiagen, Hilden, Germany), quantified in a spectrophotometer (Epoch Biotek, Agilent, Santa Clara, CA, USA), and stored at −20 °C for molecular analysis. All the manufacturer’s instructions were strictly followed. The segments of exons 5 and 7 from the *TP53* gene were amplified by a polymerase chain reaction (PCR) and analyzed by single-strand conformation polymorphisms (SSCP) and direct DNA sequencing [13].

The PCR was performed in a total volume of 25 µL, comprising 200 ng of genomic DNA, 25 pmol of sense and antisense primers, 200 µM of each dNTP, 10 mM Tris-HCl-KCl (pH8.3), 1.5 mM of MgCl2, 1U of Taq DNA polymerase platinum (Invitrogen Inc., São Paulo, SP, Brazil), and sterile water to complete the volume [13,14]. The primers used for exon 5 were previously described [13]. For exon 7, the primers were designed using Primer3Plus software (version 3.3.0) (Genbank Access: AH007665.2, June 2016) (5′CCTCATCTTGGGCCTGTGTT3′ and 5′AAATCGGTAAGAGGTGGGCC3′). The PCR was processed in a ProFlex PCR system device (Applied Biosystems by Life Technologies, Waltham, MA, USA), with an initial denaturation of 94 °C for 5 min, 35 amplification cycles at 94 °C for 1 min, annealing at 55 °C (exon 5) and 61 °C (exon 7) for 1 min, extension at 72 °C for 2 min and final extension at 72 °C for 10 min. PCR products were subjected to 2% agarose gel electrophoresis to verify the success of the reaction and then subjected to SSCP for mutation screening [13].

A non-radioactive SSCP analysis was performed according to the previously described protocol, with some modifications [13]. A total of 5 µL of PCR product was mixed with 5 µL of stop solution (0.025% bromophenol blue, 0.025% xylene cyanol, 98% formamide, 10 mM EDTA, pH 8.00), heated at 94 °C for 10 min and immediately immersed in ice. The mixture was then subjected to electrophoresis in a 7.5% polyacrylamide gel containing 0.5× TBE buffer. The run took place at 150 volts for approximately 2 h. The gels were fixed in ethanol/acetic acid and stained with 0.2% AgNO_3_. Samples that showed any difference in the electrophoretic migration of their bands were considered SSCP positive and sent for DNA sequencing [13].

The products from PCR amplifications were then subjected to direct DNA sequencing. For this, DNAs were purified using the GenElute PCR Clean-Up Kit (Sigma-Aldrich Brasil Ltd., Cajamar, Brazil), following the manufacturer’s protocol. The samples were quantified in an Epoch-Biotek spectrophotometer and diluted to 3 ng/µL. The primers used for direct sequencing were diluted to 5 pmol. The Sanger method was used for sequencing the samples according to the protocol established by the DNA Sequencing Platform of the Central Laboratory of the Federal University of Pernambuco-LABCEN/CCB/UFPE using the BigDye Terminator 3.1v kit (Thermo Fisher Scientific, Waltham, MA, USA). The samples were applied in a Genetic Analyzer ABI PRISM 3500 sequencer (Applied Biosystems). The electropherograms were evaluated using Phred PHPH-Embrapa software (http://lbi.cenargen.embrapa.br/phph, accessed on 21 June 2024) for quality analysis, and the sequences were analyzed using Seq Scanner 2 software (version 2.0) (Applied Biosystems Inc., Waltham, MA, USA) and Bioedit Sequence Alignment Editor (version 7.2, BioEdit, Informer Technhologies Inc., Los Angeles, CA, USA). The sequences were compared to the *TP53* gene reference sequence (Genbank, Accession U94788) [13,14,15].

### 2.3. Statistical Analysis

The descriptive statistical analysis was performed based on measures of central tendency, dispersion, and frequency counts. For the inferential statistical analysis of the quantitative variables, the Kolmogorov–Smirnov test was used to verify the normality of the data. Subsequently, the Student’s *t*-test was used. Frequency comparisons were obtained using the Chi-square Test. Agreement analyses were performed to verify the efficiency of the SSCP technique using the Kappa method. Spearman’s correlation coefficients (r) were classified according to Dancey and Reidy [16], as follows: r = 0.10 to 0.39 (weak), r = 0.40 to 0.69 (moderate), and r = 0.70 to 1 (strong). In all analyses, the *p*-value ≤ 0.05 was considered statistically significant. The programs used were SPSS (IBM, version 23, 2014), PRISMA (version 6.10, 2015) and GraphPad Instat (3.10, 2009).

## 3. Results

A mobility shift (SSCP positive) was found in 14.8% of Group 1 (11/74), 10% of Group 2 (4/40), and 7.5% of Group 3 (3/40) in the study of the *TP53* gene exon 5 (E5). Regarding exon 7, a mobility shift was found in 2.7% of Group 1 (2/74), 7.5% of Group 2 (3/40), and none of Group 3. The sequencing analysis for E5 showed mutations at codon 184 (GAT > AAT) in 14.8% of Group 1 (11/74), 10% of Group 2 (4/40), and 5% of Group 3 (2/40). We also observed codon 185 mutation (AGC > AGG) in 14.8% of Group 1 (11/74), 10% of Group 2 (4/40), and 7.5% of Group 3 (3/40). Patients with CME had a higher frequency of mutations in E5, than the control individuals without CME. These data show that CME may be involved with increased mutations in this exon. Between the groups that were analyzed, there was no statistically significant difference (Table 1. Around 14.8% of patients with CME presented mutations in both codons analyzed, whereas only 5% of healthy controls presented mutations involving both codons.

**Figure 1:**
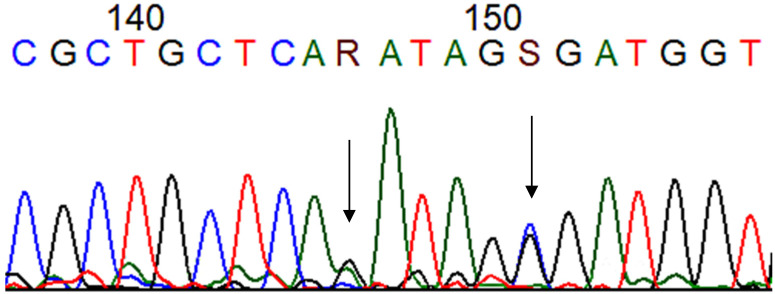
Chromatogram showing the mutations at codons 184 (R: guanine + adenine) and 185 (S: cytosine + guanine) (arrows) of the *TP53* gene after Sanger sequencing. (T: thymine, C: citosyne, A: adenine, G: guanine). Blue: cytosine, black: guanine, red: thymine and green: adenine.

Regarding exon 7, a mutation (G > T) was observed in the intronic region in 2.7% of Group 1 (2/74), 7.5% of Group 2 (3/40), and none of Group 3 (0/40). Individuals with CD had a higher frequency of mutation in E7 than those without CD. These data show that CD may be involved with increased mutations in this region. Between the groups that were analyzed, there was no statistically significant difference (Table 1). All results are shown in Figure 1 and Table 1 and Table 2.

According to the concordance analysis, our data demonstrate that SSCP is efficient in screening mutations in exon 5 and intron 7 of the *TP53* gene. The results are shown in Table 3. Regarding the megaesophagus degrees, the frequencies observed were: Grade I = 5.4% (*n* = 4), Grade II = 37.8% (*n* = 28), Grade II/III = 1.4% (*n* = 1), Grade III = 12.2% (*n* = 9), Grade III/IV = 25.7% (*n* = 19), and Grade IV = 10.8% (*n* = 8). No associations were observed among megaesophagus degrees with positive SSCP and the mutations found (Table 4).

There was a statistically significant difference between the mean ages (*p* < 0.001). This occurred because Group 3 (with negative serology for CD) was composed of young individuals who donated blood to the Blood Center of Hospital de Base in São José do Rio Preto (Table 2).

## 4. Discussion

This study investigated mutations in the *TP53* gene in patients with CME, with CD without CME, and healthy individuals. The mutations found were missense and intronic type. The mutations at codons 184 (p.D184N) and 185 (p.S185R) were heterozygous. The p.D184N mutation leads to substituting the amino acid aspartate for asparagine. This somatic mutation was reported in 28 types of tumors, and it is considered partially functional concerning the transcriptional activity of the p53 protein [17,18]. The p.S185R mutation leads to the substitution of the amino acid serine for arginine, and it is located in CpG islands. It has already been described in breast cancer and is considered functional concerning the transcriptional activity of the p53 protein [18,19]. The mutation found in intron 7 is a transition G:C > T:A and does not change the structure of the protein [20]. These mutations are not associated with megaesophagus degrees. Such mutations are located in the *TP53 DNA-binding* domain, featuring a gain of function, which favors the development of malignant phenotypes [10].

Manoel-Caetano and colleagues evaluated the presence of mutations in individuals with CME and concluded that mutations in the *TP53* gene are rare events in the esophageal mucosa of patients with CD [21]. Lacerda and colleagues found mutations in 40.6% of patients with both CME and ESCC diseases, in 45% of the non-Chagasic ESCC, and 3% of the benign CME. Missense mutations were the most common in the three groups. The two groups with ESCC were associated with high alcohol and tobacco consumption, older age, and worse prognosis compared to the CME group. These authors concluded that the high frequency of mutations found in the esophageal tissue of these individuals suggested that the *TP53* gene plays an essential role in the tumorigenic process of patients with CME [14].

Martins and colleagues analyzed patients with ESCC and CME who had lower smoking habits. This fact showed that achalasia is the predominant risk factor for the development of cancer in these patients. The authors concluded that patients with EC associated with megaesophagus have a poor prognosis if the tumor is not recognized early [22].

In the present study, the SSCP technique was efficient in screening mutations in exon 5 and intron 7 of the *TP53* gene. SSCP analysis allows tracking 50–100% of mutations in fragments up to 500 bp. Differences in ssDNA mobility are usually due to conformational variations of the molecules. This conformation is affected by factors such as gel temperature during electrophoresis, running buffer concentration, and the presence of denaturing agents. Higher temperatures can destroy some semistable conformations [23]. Some recent studies have shown that *TP53* variants can be detected in the circulating DNA from the blood plasma of breast and ovarian cancer patients [24,25,26]. In this study, we were able to detect *TP53* gene mutations in the circulating DNA from the blood plasma of patients with CD.

Silveira et al. [13] observed mutations in exons 5 and 6 of the *TP53* gene (codons 147 and 197) in 22% of patients with ESCC, but no mutation was found in patients with CE. The authors concluded that *TP53* gene mutations are not common in CE but are frequent in ESCC [13]. A recent study revealed that the most frequently mutated gene in ESCC was *TP53* (96.6%, 28/29) [27], while a recent systematic review and meta-analysis revealed that *TP53* (68.6%; 95% CI: 61.6–74.9) was the genetic factor most commonly involved with ESCC [28].

Our study showed, for the first time, simultaneous mutations at codons 184 and 185 of the *TP53* gene in patients with CME (Stages I to IV) and Chagasic patients without megaesophagus. In conclusion, this study shows that *TP53* mutations are frequent in patients with CD. More studies are needed to assess whether the simultaneous presence of mutations at codons 184 and 185 increases the risk of developing ESCC in these patients.

### Limitations of the Study

The age difference between the healthy control group and the other groups occurred because the authors obtained samples from blood donors from the blood bank, which are made up of younger individuals.

## Figures and Tables

**Table 1:** Demographic characteristics and frequencies of mutations in patients with CME (G1), without CME (G2) and control individuals (G3).

	G1 *N* = 74 (%)	G2 *N* = 40	G3 *N* = 40	*p*-Value
Mean Age (± SD)	67.03 ± 11.09	75.95 ± 12.20	46.32 ± 11.00	*p* < 0.001
Max	64.44	71.99	42.06	
Median	67.00	78.00	44.50	
Min	42.00	43.00	29.00	
Female	41 (56.16)	23 (58.97)	14 (36.84)	*p* = 0.162
Male	32 (43.84)	16 (41.03)	24 (63.16)	
Mut pD184N (%)	11 (14.8%) ^a^	4 (10%) ^b^	2 (5%) ^c^	*p* = 0.56 ^a,b^ *p* = 0.21 ^a,c^ *p* = 0.67 ^b,c^
Mut pS185R (%)	11 (14.8%) ^a^	4 (10%) ^b^	3 (7.5%) ^c^	*p* = 0.56 ^a,b^ *p* = 0.37 ^a,c^ *p* = 1.00 ^b,c^
Mut G > T intron 7 (%)	3 (4%) ^a^	3 (7.5%) ^b^	0 ^c^	*p* = 0.32 ^a,b^ *p* = 1.00 ^a,c^ *p* = 0.23 ^b,c^

*p*-value calculated by chi-square test; SD: standard deviation; ^a,b^ = chi-square test comparing groups G1 and G2; ^a,c^ = chi-square test comparing groups G1 and G3; ^b,c^ = chi-square test comparing groups G2 and G3.

**Table 2:** Characterization of the mutations found according to Bioedit [15] and the IARC TP53 Database [17].

Codons	Type	Classification	Aa *	Transcriptional Activity	SIFT Class ^§^	Splice Site	CpG Site	Hot Spot	DNA Binding
184	G:C > A:T	Transition	Asp > Asn	Partially Functional	Tolerant	-		No	Yes
185	C:G > G:C	Transversion	Ser > Arg	Functional	Tolerant	-	Yes	No	Yes
Intron 7	G:C > T:A	Transition				No	No	No	

* Aa: amino acid; Val: valine; Leu: leucine; Asp: aspartic acid; Asn: asparagine; Ser: serine; Arg: arginine. ^§^ SIFT class: sort intolerant from tolerant.

**Table 3:** Concordance analysis showing the efficiency of SSCP in screening mutations at the *TP53* gene.

	Kappa Values	Significancy	Pearson Qui-Square	*p*-Value
SSCP vs. Mutation 184—exon 5	0.97	0.00	142	0.00
SSCP vs. Mutation 185—exon 5	1.00	0.00	154	0.00
SSCP vs. Mutation—intron 7	1.00	0.00	308	0.00

**Table 4:** Association between the degree of megaesophagus and the identified mutations.

	Grade I N(%)	Grade II	Grade II/III	Grade III	Grade III/IV	Grade IV	*p*-Value
pD184N pos	1	4 (14%)	0	2	3 (15.8%)	1	0.97
pD184N neg	3	24	1	7	16	7	
Overall	4	28	1	9	19	8	
pS185R pos	1	4 (14%)	0	2	3 (15.8%)	1	0.97
pS185R neg	3	24	1	7	16	7	
Overall	4	28	1	9	19	8	
G > T intron 7 pos	0	2 (7%)	0	0	0	0	0.69
G > T intron 7 neg	4	26	1	9	19	8	
Overall	4	28	1	9	19	8	

*p*-value calculated by chi-square test.

## Data Availability

The original contributions presented in the study are included in the article, further inquiries can be directed to the corresponding author.
